# Neural Systems Under Change of Scale

**DOI:** 10.3389/fncom.2021.643148

**Published:** 2021-04-21

**Authors:** Erik D. Fagerholm, W. M. C. Foulkes, Yasir Gallero-Salas, Fritjof Helmchen, Karl J. Friston, Robert Leech, Rosalyn J. Moran

**Affiliations:** ^1^Department of Neuroimaging, King’s College London, London, United Kingdom; ^2^Department of Physics, Imperial College London, London, United Kingdom; ^3^Brain Research Institute, University of Zürich, Zurich, Switzerland; ^4^Neuroscience Center Zurich, Zurich, Switzerland; ^5^Wellcome Centre for Human Neuroimaging, University College London, London, United Kingdom

**Keywords:** scalable neural systems, scale free neural systems, mechanical similarity, dynamic causal modeling (DCM), computational neuroscience, theoretical neuroscience, renormalisation group theory

## Abstract

We derive a theoretical construct that allows for the characterisation of both scalable and scale free systems within the dynamic causal modelling (DCM) framework. We define a dynamical system to be “scalable” if the same equation of motion continues to apply as the system changes in size. As an example of such a system, we simulate planetary orbits varying in size and show that our proposed methodology can be used to recover Kepler’s third law from the timeseries. In contrast, a “scale free” system is one in which there is no characteristic length scale, meaning that images of such a system are statistically unchanged at different levels of magnification. As an example of such a system, we use calcium imaging collected in murine cortex and show that the dynamical critical exponent, as defined in renormalization group theory, can be estimated in an empirical biological setting. We find that a task-relevant region of the cortex is associated with higher dynamical critical exponents in task vs. spontaneous states and vice versa for a task-irrelevant region.

## Introduction

### Scalable Dynamical Systems

Let us consider a dynamical system that is evolving in time and generating a certain series of states. If we now change the size of the system, it will begin generating states that are different from those of the original unscaled system. Note that by changing the “size” of a system, we refer to a transformation that alters the spatial coordinate of the governing equation of motion in question. For instance, when we speak of varying the size of a planetary orbit, we do not allude to the size of the planet itself, but rather to the spatial coordinate of its centre of mass as it orbits its host star. Upon making such a transformation, we refer to the system as being “scalable” if the equations of motion describing both its scaled and unscaled versions are identical in form – a system that Landau referred to as possessing “mechanical similarity” (Landau and Lifshitz, 1976).

As an example of a scalable system, we consider the trajectory *r*(*t*) of a planet orbiting a sun, which is found via Newton’s second law:

(1) $$m\,\frac{d^{2}\,\left[r\,\left(t\right)\right]}{d\,t^{2}}=-\frac{G\,M\,m}{r^{3}\,\left(t\right)}\,r\,\left(t\right),$$

Where *m* is the mass of the planet, *M* is the mass of the sun, and *G* is the universal gravitational constant.

We now transform the planet’s trajectory *r*(*t*) to a scaled trajectory*r*_*scaled*_(*t*) as follows:

(2) $$r\,\left(t\right)\to r_{\mathrm{scaled}}\,\left(t\right)≜b\,r\,\left(b^{\alpha }\,t\right),$$

Where *b* is an arbitrary scale factor and α is a constant to be determined.

In order to show that planetary motion is scalable, we must demonstrate that if *r*(*t*) is a solution of the equation of motion in Eq. (1), then *r*_*scaled*_(*t*) must be a different, but equally valid, solution.

To find the equation of motion satisfied by the scaled trajectory *r*_*scaled*_(*t*) we begin by replacing the independent variable *t* with a scaled version of the independent variable *b*^α^*t* in Eq. (1) such that:

(3) $$m\,\frac{d^{2}\,\left[r\,\left(b^{\alpha }\,t\right)\right]}{d\,{\left(b^{\alpha }\,t\right)}^{2}}=-\frac{G\,M\,m}{r^{3}\,\left(b^{\alpha }\,t\right)}\,r\,\left(b^{\alpha }\,t\right),$$

or equivalently:

(4) $$m\,\frac{d^{2}\,\left[b\,r\,\left(b^{\alpha }\,t\right)\right]}{d\,t^{2}}=-b^{2\,\alpha +3}\,\frac{G\,M\,m}{{\left(b\,r\,\left(b^{\alpha }\,t\right)\right)}^{3}}\,b\,r\,\left(b^{\alpha }\,t\right),$$

which, using Eq. (2) can be written as:

(5) $$m\,\frac{d^{2}\,\left[r_{\mathrm{scaled}}\,\left(t\right)\right]}{d\,t^{2}}=-b^{2\,\alpha +3}\,\frac{G\,M\,m}{r_{\mathrm{scaled}}^{3}\,\left(t\right)}\,r_{\mathrm{scaled}}\,\left(t\right),$$

where the *b*^2α + 3^ factor on the right-hand side of Eq. (5) prevents the scaled trajectory, *r*_*scaled*_(*t*), from satisfying Newton’s second law in Eq. (1). Instead, the scaled trajectory describes the motion of a planet orbiting a sun with a different mass: *M*_*scaled*_ = *b*^2α + 3^*M*. However, if we choose a value of α that allows for *M*_*scaled*_ = *M*, which occurs when:

(6) 2⁢α+3=0⇒α=-3⁢/⁢2,

then the equation of motion for the scaled trajectory *r*_*scaled*_(*t*) becomes identical in form to the equation of motion for the original trajectory *r*(*t*). The value of α in Eq. (6) shows us that if *r*(*t*) is a solution, then so is *r*_*scaled*_(*t*)≜*br*(*b*^−3/2^*t*) for any choice of scaling parameter *b*. This demonstrates that Newton’s second law is scalable if the square of the period of the orbit is proportional to the cube of its semi-major axis, i.e., Kepler’s third law.

We simulate orbits of increasing size and show that one can recover Kepler’s third law from simulated data using Parametric Empirical Bayes (PEB) (Friston et al., 2016). The latter is a hierarchical statistical model that rests on the principles of dynamic causal modelling (DCM) and uses variational Bayes to estimate the strength of effective connectivity between the orbiting bodies at different scales.

### Scale Free Dynamical Systems

We defined a scalable system above as one in which a change in size produces a new state (the scaled orbit) which is different (larger/smaller with a longer/shorter orbital period), but one that is an equally valid solution of the equation of motion. In contrast, a scale free system is *itself* invariant under transformation of scale. This means that zooming in or out leaves images of the states statistically unchanged as observed, for example, in systems exhibiting turbulent flow (Bohr, 1998).

Scale freeness is of considerable interest in neuroscience due to increasing evidence that the brain exhibits scale freeness across several orders of magnitude, ranging from single-cell recordings (Beggs and Plenz, 2003), to meso-scale circuits (Scott et al., 2014) and entire brain regions (He, 2011). Studies in this field often address power law distributions of graph theoretic metrics (Eguiluz et al., 2005) or of probability distributions of cascading events (Tagliazucchi et al., 2012). However, these metrics are often inadequate due to the limited spatiotemporal extent of the techniques and are therefore unable to rigorously characterize dynamics of different brain states. Scale free dynamics, in systems with both long and short-range interactions, are associated with a divergence in correlation length (Beggs and Timme, 2012) – a characteristic that is thought to provide functional benefits within neural systems (Shew and Plenz, 2013). One particularly prominent area of research lies in the study of scale free properties of temporal fluctuations in neural activity, which can be quantified by, for example, the Hurst exponent – calculated via detrended fluctuation analysis (DFA) (Ros et al., 2017; Dong et al., 2018).

We demonstrate a link between scalable and scale free systems, as shown by a relationship between temporal rescaling (in scalable systems) and the dynamical critical exponent (in scale free systems). Using the same basic methodology employed for systems varying in size, we then characterize scale freeness using coarse graining – a recently active area of research in the context of neural systems (Agrawal et al., 2019; Meshulam et al., 2019; Nicoletti et al., 2020). We use calcium imaging data collected in a murine model with high spatiotemporal resolution (∼40 μm, 50 ms) to show that dynamical critical exponents are higher in the task state than in the spontaneous state in task-relevant regions of the cortex and vice versa in task-irrelevant regions.

In summary, we derive a theoretical construct and accompanying methodology that allows for the characterisation of both scalable and scale free systems within the same DCM-based framework.

## Materials and Methods

### The DCM Recovery Model

The DCM recovery model is given by:

(7) $$\frac{d\,\left[r\,\left(t\right)\right]}{d\,t}=A\,r\,\left(t\right)+C\,v\,\left(t\right)+\omega^{\left(r\right)}\,\left(t\right),$$

where we refer the reader to the original DCM paper (Friston et al., 2003) for a full derivation; *r* is a column vector representing the state of the system in question; *A* is the intrinsic coupling matrix. The term “intrinsic” indicates that this coupling matrix mediates system states that are intrinsic to the system. In linear state space models this would be the system matrix that plays the role of a Jacobian; *C* is the extrinsic connectivity matrix; *v* = *u* + ω^(*v*)^, where ω^(*v*)^ is a noise term describing random, non-Markovian fluctuations on external perturbations *u*; and ω^(*r*)^ is a *n*-component column vector of noise terms describing random, non-Markovian fluctuations on *r* (Li et al., 2011). If *r* has *n* components and there are *m* perturbing inputs *v*, then *A* is a *n*×*n* matrix and *C* is a *n*×*m* matrix. Note that, although all numerical methods used here accommodate noise via ω^(*r*)^, we omit this term henceforth for the sake of clarity.

Using Eq. (7), we are able to model arbitrary dynamical systems in a way that allows for an attended inversion procedure which, given data, enables us to estimate the underlying model parameters in the presence of noise in states and measurements. This inverse scheme is a crucial aspect of the methodology, as it enables an estimation of the ways in which an arbitrary dynamical system is connected – both intrinsically and extrinsically.

Note that Eq. (7) is the form of the equation of motion we use for all Bayesian model inversions in this paper and we provide publicly available MATLAB code to allow for reproduction of results, as well as for analyses of timeseries of arbitrary dimensionality from any neuroimaging modality.

### Scalability in the DCM Recovery Model

Here, we derive the conditions that allow for the DCM recovery model in Eq. (7) to be scalable. As with Newton’s second law, we would like to transform Eq. (7) according to Eq. (2), and to this end we begin by replacing *t* with *b*^α^*t* in Eq. (7), such that:

(8) $$\frac{d\,\left[r\,\left(b^{\alpha }\,t\right)\right]}{d\,\left(b^{\alpha }\,t\right)}=A\,r\,\left(b^{\alpha }\,t\right)+C\,v\,\left(b^{\alpha }\,t\right),$$

or equivalently:

(9) $$\frac{d\,\left[b\,r\,\left(b^{\alpha }\,t\right)\right]}{d\,t}=b^{\alpha }\,A\,b\,r\,\left(b^{\alpha }\,t\right)+b^{\alpha +1}\,C\,v\,\left(b^{\alpha }\,t\right),$$

which, using Eq. (2), can be written as:

(10) $$\frac{d\,\left[r_{\mathrm{scaled}}\,\left(t\right)\right]}{d\,t}=b^{\alpha }\,A\,r_{\mathrm{scaled}}\,\left(t\right)+b^{\alpha +1}\,C\,v\,\left(b^{\alpha }\,t\right),$$

which differs from Eq. (7) for all values of α. Therefore, as opposed to Newton’s second law, it is not possible to render the original Eq. (7) identical in form to the scaled Eq. (10) simply by specifying a value of α. Instead, scalability [such that *r*_*scaled*_(*t*)≜*br*(*b*^α^*t*) becomes a possible solution] requires that the parameters of the DCM recovery model in Eq. (7) also change relative to system size.

Specifically, we require that the frequency of external perturbations transform as follows:

(11) $$v\,\left(t\right)\to v_{\mathrm{scaled}}\,\left(t\right)=v\,\left(b^{\alpha }\,t\right),$$

which allows us to write Eq. (10) as:

(12) $$\frac{d\,\left[r_{\mathrm{scaled}}\,\left(t\right)\right]}{d\,t}=b^{\alpha }\,A\,r_{\mathrm{scaled}}\,\left(t\right)+b^{\alpha +1}\,C\,v_{\mathrm{scaled}}\,\left(t\right).$$

Furthermore, the elements of the connectivity matrices must transform as follows:

(13) $$\begin{array}{l} a_{ij}\to \frac{b_{i}^{\text{α}+1}}{b_{j}}a_{ij},\\ c_{ij}\to \frac{b_{i}^{\text{α}+2}}{b_{j}}c_{ij}, \end{array}$$

where *a*_*ij*_ is the intrinsic coupling between the *i*th and *j*th regions; *b*_*i*_ is the factor by which the *i*th node is scaled; *b*_*j*_ is the factor by which the *j*th node is scaled; and *c*_*ij*_ is the extrinsic coupling between the *i*th and *j*th regions.

However, for the cases discussed in this paper (in terms of intrinsic connectivity only), all nodes are scaled by equal amounts, which means that we use the following simplified versions of Eq. (13):

(14) $$\begin{array}{l} A\to A_{scaled}=b^{\text{α}}A,\\ C\to C_{scaled}=b^{\text{α}+1}C. \end{array}$$

We then use Eq. (14) to write Eq. (12) as:

(15) $$\frac{d\,\left[r_{\mathrm{scaled}}\,\left(t\right)\right]}{d\,t}=A_{s\,c\,a\,l\,e\,d}\,r_{\mathrm{scaled}}\,\left(t\right)+C_{s\,c\,a\,l\,e\,d}\,v_{\mathrm{scaled}}\,\left(t\right),$$

which we see is now identical in form to Eq. (7), thus achieving scalability in the DCM recovery model.

### Orbital Mechanics Simulation

We simulate three bodies (one sun and two planets) orbiting a common centre of gravity, using a modified version of a freely available *n*-body physics simulator, as part of the Unity3D gaming engine (version 2017.3.1f1) (Unity Technologies, 2017). The mass of the star is 10^5^ times greater than that of the two planets. This is sufficiently massive so that the wobble of the sun about the centre of gravity of the three-body system is zero to within-software precision. We begin with a simulation in which the semi-major axes of the orbits of the two planets differ by 10%. We run this simulation a total of ten times with the same initial conditions, except that in each new simulation we increase the sizes of both semi-major axes by 10%. This allows us, using first-level DCM models, to obtain estimates of the intrinsic connectivity (gravitational attraction) in the system for each of the ten orbital sizes (see Appendix 1).

We then perform second-level hierarchical modelling (PEB) (see Appendix 2) in order to characterise the change in intrinsic connectivity from data collected in orbits of different sizes. This allows us to recover the value of α in Eq. (2) with the highest model evidence by assuming that the planetary trajectories: (a) can be approximated by solutions of the DCM recovery model in Eq. (7); and (b) are known *a priori* to be scalable. In this way, we are able to test whether the highest model evidence for the theoretically predicted relationships between intrinsic connectivity and scale in Eq. (14) is obtained when α lies close to the value known *a priori* from Kepler’s third law in Eq. (6).

### Coarse Graining Neuroimaging Data

So far, we have considered scaling operations in terms of changes to the physical size of a system, using the example of orbital motion. However, in neuroimaging we face a different situation, in which data is collected at a single scale. Therefore, rather than changing physical size, we now perform scaling operations in terms of coarse graining, i.e., we change the resolution at which neuroimaging data is observed.

Throughout the analyses presented, we perform coarse graining by repeating the following two steps as many times as an image will allow: (a) We combine 2 × 2 neighbouring “regions” of an image into “blocks”. The timecourse of a given block is defined as the mean of the timecourses of its constituent regions; (b) We then redefine regions such that each one now occupies the same spatial extent of the image as a block occupied in step (a). Similarly, we redefine blocks such that each one now consists of the newly defined (larger) 2 × 2 regions.

### Scale Freeness in Neuroimaging Data

In the orbital mechanics example, we used the dependent variable *r* to refer to the position of a given planet. In dealing with neuroimaging data, we now instead use *x* and *X* to refer to the measured signal intensities of a given region and block, respectively. We have no reason to assume a linear change in measured signal intensity between averaged timecourses from progressively larger portions of an image. We must therefore alter Eq. (2) to include a new scaling exponent β, such that:

(16) $$x\,\left(t\right)\to x_{\mathrm{scaled}}\,\left(t\right)≜b^{\beta }\,x\,\left(b^{\alpha }\,t\right).$$

However, in all subsequent analyses we deal with timecourses that have zero mean and unit variance, meaning that we can use the following simplified version of Eq. (16):

(17) $$x\,\left(t\right)\to x_{\mathrm{scaled}}\,\left(t\right)≜x\,\left(b^{\alpha }\,t\right).$$

Therefore, the dynamics of a system are scale free if (on average) the following relationship between timecourses of regions *x*(*t*) and blocks *X*(*t*) holds:

(18) $$X\,\left(t\right)=x\,\left(b^{\alpha }\,t\right).$$

It is this relationship that we test using the DCM recovery model in Eq. (7).

### Scaling and Renormalization in DCM

We now examine the way in which coarse graining affects characteristic relaxation times in blocks and their constituent regions. This relationship is encoded in the dynamical critical exponent *z*.

We begin by summing over a total of *N* regions and normalizing to define the region-wise characteristic decay time *t*_*r*_ as the time taken for the time correlation function *C*_*r*_(*t*):

(19) $$C_{r}\,\left(t\right)=\frac{1}{N}\,\sum_{i=1}^{N}x_{i}\,\left(t\right)\,x_{i}\,\left(0\right),$$

to decay to 1e of its initial value:

(20) $$C_{r}\,\left(t_{r}\right)=\frac{C_{r}\,\left(0\right)}{e}.$$

Similarly, we sum over a total of $\frac{N}{b^{2}}$ blocks, as each block consists of 2 × 2 regions, and normalize to define the block-wise characteristic decay time *t*_*b*_ as the time taken for the time correlation function *C*_*b*_(*t*):

(21) $$C_{b}\,\left(t\right)=\frac{b^{2}}{N}\,\sum_{I=1}^{\frac{N}{b^{2}}}X_{I}\,\left(t\right)\,X_{I}\,\left(0\right),$$

to decay to 1e of its initial value:

(22) $$C_{b}\,\left(t_{b}\right)=\frac{C_{b}\,\left(0\right)}{e}.$$

Note that we assume that the correlation functions in Eqs (19) and (20) decay over time due to the fact that the intrinsic coupling matrix A in the governing equation of motion (7) is given negative priors along its main diagonal. The eigenvalues of A therefore have negative real components, which means that the resulting dynamics are situated in the stable top-left quadrant of the trace-determinant plane, in which timeseries decay following perturbation.

If the system is scale free, then using Eqs (18), (19), and (21) we see that:

(23) $$C_{b}\,\left(t\right)=C_{r}\,\left(b^{\alpha }\,t\right).$$

If we then choose *t* = *b*^−α^*t*_*r*_ then using Eqs (20), (22), and (23) we see that:

(24) $$C_{b}\,\left(b^{-\alpha }\,t_{r}\right)=C_{r}\,\left(t_{r}\right)=\frac{C_{r}\,\left(0\right)}{e}=\frac{C_{b}\,\left(0\right)}{e},$$

where $\frac{C_{b}\,\left(0\right)}{e}$ is the definition of the block time scale *t*_*b*_ from Eq. (22) and hence:

(25) $$t_{b}=b^{-\alpha }\,t_{r}.$$

In Renormalization Group (RG) theory, the dynamical critical exponent *z* is defined as follows:

(26) $$t_{b}=b^{z}\,t_{r},$$

which, together with Eq. (25), shows us that:

(27) α=-z.

Therefore, in the context of coarse graining, by estimating α, we are in fact estimating the negative dynamical critical exponent, such that the transformation of connectivities in Eq. (14) can be re-formulated as:

(28) $$\begin{array}{l} A\to A_{scaled}=b^{-z}A,\\ C\to C_{scaled}=b^{1-z}C, \end{array}$$

thereby creating a link, via Eq. (27), between scalable [see Eq. (14)] and scale free [see Eq. (28)] systems via the connectivities in the system. Note that the term “connectivity” here is not to be confused with the way in which the term is used in in RG theory. Rather, the connectivities here describe interaction strengths (i.e., effective connectivity) between either: (a) elements that vary in distance between one another in scalable systems; or (b) regions within blocks of increasing size within an image. Note also that the DCM in Eq. (7) is a linear approximation of a generic non-linear dynamical system, which could in principle be extended using a Taylor series expansion (Stephan et al., 2008). In this case, the B and D matrices used in higher-order DCMs both form part of the Jacobian and, as such, must transform exactly in the same way as the intrinsic A matrix does in scalable systems as in Eq. (14) and in scale free systems as in Eq. (28).

As with the orbital example, we then perform PEB modelling in order to characterise the change in intrinsic connectivity from calcium imaging data collected in murine cortex at different levels of coarse graining. This allows us to recover the dynamical critical exponent *z* with the highest model evidence by assuming that neural timeseries: (a) can be approximated by solutions of the DCM recovery model in Eq. (7); and (b) are known *a priori* to be scale free – an assumption that is justified by the empirical evidence, albeit under debate, indicating the presence of scale free dynamics in neural systems (Breakspear, 2017; Zhigalov et al., 2017; Palva and Palva, 2018).

## Results

### Orbital Simulation

**FIGURE 1:**
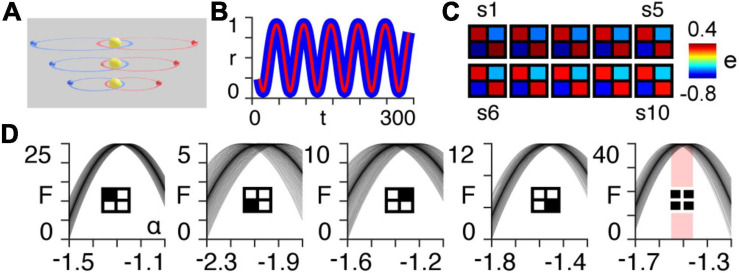
Orbital simulation. **(A)** The three-body system orbiting a sun at different scales. Note that in the results we use a total of ten scales. **(B)** Normalized radial distance of the first planet from the centre of mass of the three-body system at the largest scale, as a function of time for the first 300 timepoints of the simulation. The blue line is the true trajectory obtained from the simulation and the red line is the estimated trajectory following Bayesian model inversion. **(C)** A posteriori estimates (e) of coupling strength (gravitational attraction) following first-level modelling (see Appendix 1) of the three-body system for each of the ten orbital scales (s1–s10). **(D)** Approximate lower bound log model evidence given by the free energy (see Appendix 1), following second-level modelling of the ten scales shown in panel **(B)**, as a function of temporal rescaling α. Each curve corresponds to one of the 100 trials in which Gaussian noise is added to the scaling parameter in order to obtain a distribution of peak free energies (see Appendix 2). The first four panels (from left to right) pertain to the individual intrinsic coupling matrix elements, as indicated by the insets. The fifth column shows the free energies summed across the four individual matrix elements. The red bar indicates the range of peak free energies.

Here, we simulate the motion of two planets orbiting a sun for ten different scales, each with orbital paths of progressively larger semi-major axes (Figure 1A).

Using Bayesian model inversion, we recover estimates of both the orbital trajectories (Figure 1B), as well as the intrinsic connectivity matrices associated with each scale (Figure 1C). We then use hierarchical modelling (PEB) to assess the extent to which the intrinsic connectivity transformation in Eq. (14) can explain variability across orbital paths of different sizes, for a range of power law exponents α (see Appendix 2). The peak log model evidence (see Appendix 1) for the entire two-body system (Figure 1C, last column on right) is found to be approximately distributed around α = −32 for 100 trials containing noise (see Appendix 2), as predicted by Kepler’s third law in Eq. (6) (Figure 1D).

### Neuroimaging Data

**FIGURE 2:**
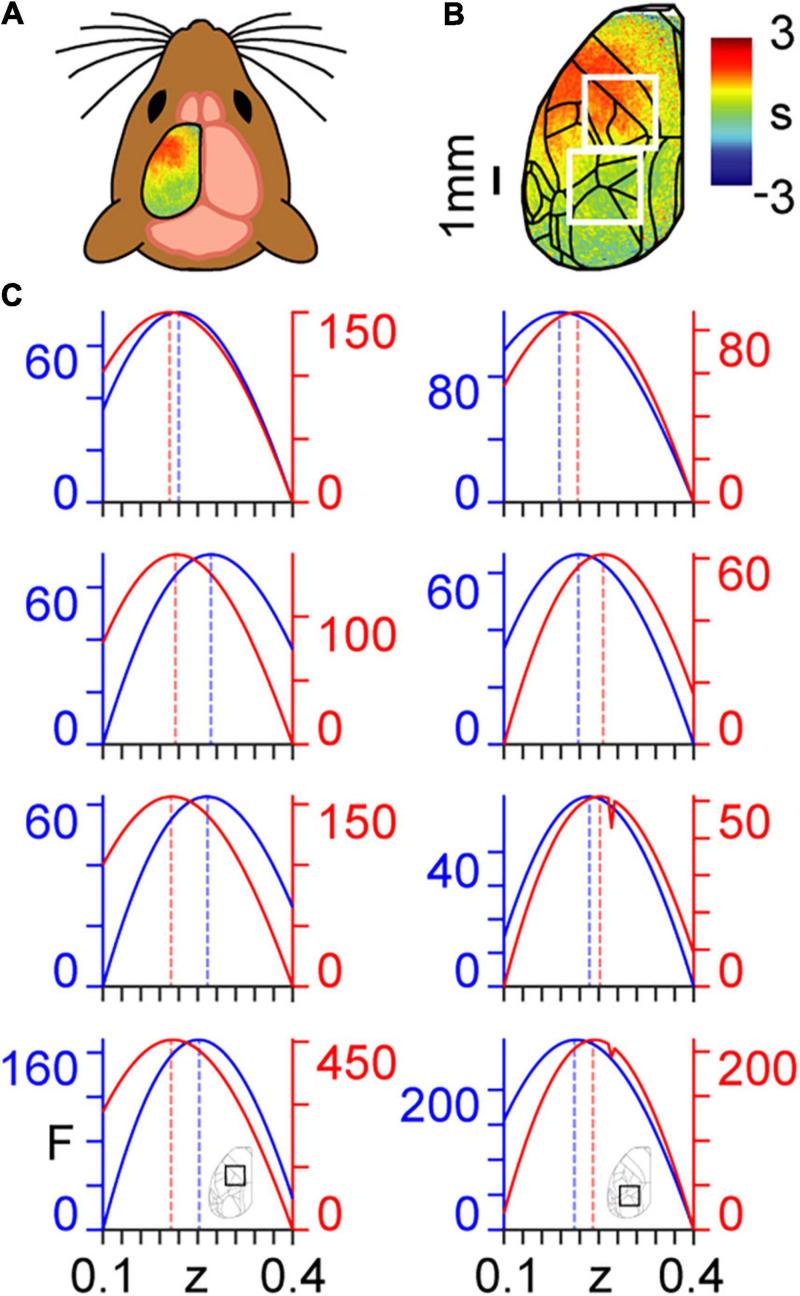
Coarse graining of calcium imaging data: **(A)** wide-field calcium imaging over the left hemisphere of a head-fixed mouse, expressing GCaMP6f in layer 2/3 excitatory neurons. **(B)** Example z-scored (DF/F) activity averaged over a 10 s trial length, shown as standard deviation (s) of the signal from the mean. Cortical areas are aligned to the Allen Mouse Common Coordinate Framework. The top and bottom white squares correspond to ROIs 1 and 2, respectively. **(C)** Approximate lower bound log model evidence given by the free energy (F) as a function of the dynamical critical exponent (z), following PEB modelling across coarse-grained scales for spontaneous (blue) and task (red). Maximum values are indicated by the dashed vertical lines. Results in the left and right columns correspond to ROIs 1 and 2 in panel **(B)**, respectively, as shown by the insets in the bottom row. Free energy values are presented individually for the three mice (rows 1–3 from top to bottom) and summed across the three mice (row 4, bottom).

Here, we use a coarse graining approach to determine the dynamical critical exponents associated with calcium imaging data collected in mice (Figures 2A,B) that are either in an awake resting state (spontaneous activity) or performing a task (Helmchen and Gallero-Salas, 2020). We show the ways in which the calcium imaging data and intrinsic connectivity matrix transform between scales in Supplementary Movie 1 and Supplementary Figure 1, respectively. Results are presented for *n=3* mice, with analyses performed separately within two regions of interest (ROIs) (Figure 2C). ROI 1 (the top white square in Figure 2B) covers principally forelimb, hindlimb, and motor cortices; i.e., areas not directly involved in the task. ROI 2 (the bottom white square in Figure 2B) covers principally posterior parietal and visual cortices; i.e., areas directly involved in the task (see Appendix 3). We note three main results with reference to Figure 2C, which were remarkably consistent over the three mice analysed:

(a) All values of the dynamical critical exponent *z* are positive, which indicates, via Eq. (26), that signal fluctuations decay more slowly in larger cortical structures.
(b) The dynamical critical exponent *z* that maximises model evidence is higher in the spontaneous state in the task-irrelevant region (ROI 1).
(c) The dynamical critical exponent, *z*, that maximises model evidence is higher in the task state in the task-relevant region (ROI 2).

## Discussion

Let us imagine making a copy of a dynamical system that is identical to the original in every way, except that it is twice its size. What we observe is that, purely by virtue of their difference in size, the two systems will generate entirely different states. However, in certain cases it is possible to compensate for such a change in size through transformations to other model parameters which, in turn, allows for both the original and the larger version to be governed by the same equation of motion. We refer to systems that possess this property of single equation governance as being “scalable”. In this paper, we derive the precise ways in which parameters must transform, relative to system size, in order for the DCM recovery model to be scalable. We then translate these theoretical transformations into a methodology for determining, via hierarchical modelling, the temporal rescaling factor associated with the highest model evidence for any scalable system.

A planetary trajectory is an example of a scalable system, due to the fact that an orbit that is increased in size will result in an entirely new orbit – but one that is an equally valid solution of Newton’s second law. In addition, we know that the scalability of planetary motion requires the square of the period of the orbit to be proportional to the cube of its semi-major axis, i.e., Kepler’s third law. This additional fact allows for planetary motion to be used, not only as a known case of scalability, but also as a ground truth model with a temporal rescaling factor known *a priori* from Kepler’s third law. Specifically, one can show that the highest variational free energy (model evidence) obtained from Bayesian model inversion of orbital timeseries is associated with the Kepler factor – thus providing construct validation.

**FIGURE 3:**
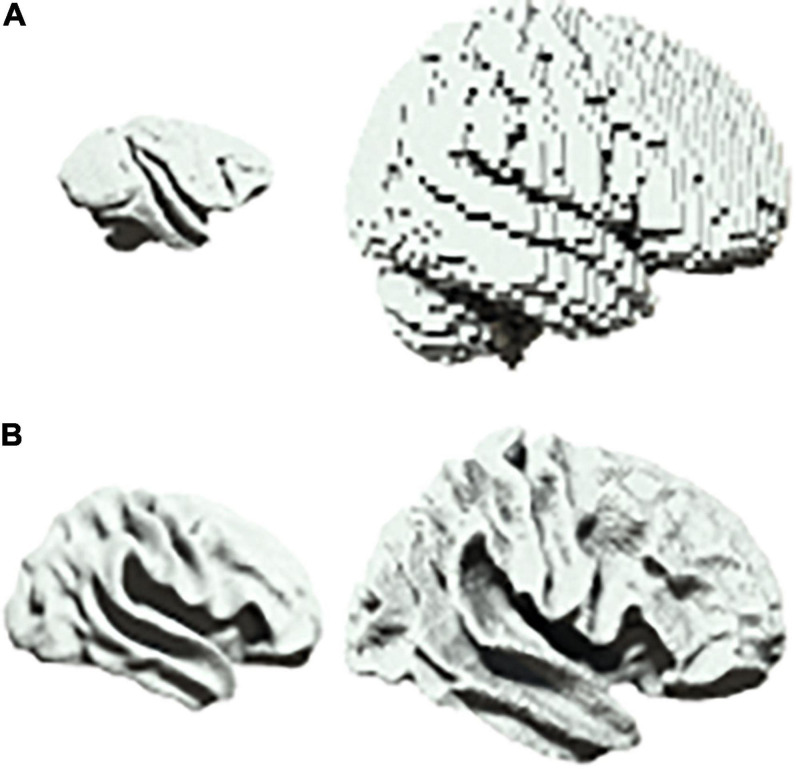
Scalability in neural systems. **(A)** Vervet monkey (left) and human (right). **(B)** Inflated cortical surfaces from an infant (left) and adult (right) human.

There are a range of scenarios in biology in which one may wish to characterize scalable system architectures, for example in the phylogenetic or ontogenetic scaling of neural structures (Buzsaki et al., 2013). In neuroimaging, one would usually account for differences in size by first projecting data into a common space before beginning the analysis of neural dynamics (Muller et al., 2018). For example, homologues are first identified between brain regions when comparing different species such as primates and humans (Figure 3A). Similarly, when comparing across development, infant and adult brains are first aligned onto a common template (Figure 3B).

Let us now go back to our original dynamical system and, rather than making a new scaled copy, imagine that we instead zoom in to twice the level of magnification within the same system. What we see is that the states generated at these two levels are completely different. However, there are certain cases in which it is impossible to tell, due to a lack of characteristic length scale, whether any magnification has in fact taken place – we refer to such systems as being “scale free”.

**FIGURE 4:**
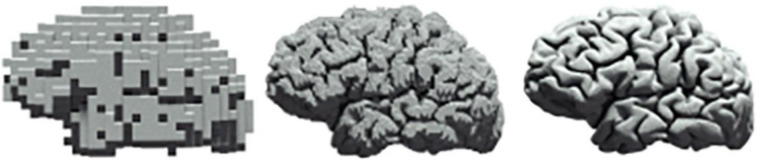
Scale freeness in neural systems. The same human brain at three different levels of resolution.

Investigations in neuroscience tend to be reported in isolation, e.g., either at the cellular level (Ouzounov et al., 2017) or at the level of a large population of neurons (Power et al., 2017), with microscopic findings seldom being translated to macroscopic properties of neural circuits. As such, there is continuing interest within theoretical (Di Santo et al., 2018), computational (Ly et al., 2019), as well as experimental (Cocchi et al., 2017) neuroscience in the characterisation of scale free dynamics in neural systems (Figure 4). In contrast, we propose a methodology in this paper that allows for a unified approach to the quantification of cross-scale dynamics.

We show that there is a connection between scalable and scale free systems in terms of the derived DCM model parameter transformations. It is this connection that allows us, via the same basic methodology used to recover the Kepler factor in planetary motion, to recover the state-dependent dynamical critical exponents in different regions of murine cortex. Specifically, we show that the dynamical critical exponent is higher in a task state (as compared with a spontaneous state) in a task-relevant region and vice versa for a task-irrelevant region of the cortex. The study of critical dynamics remains an ongoing area of research in neural systems (Plenz and Thiagarajan, 2007; Yu et al., 2011; Wu et al., 2019), as these have been associated with functional benefits in the operation of the cortex, such as allowing for the conditions necessary for optimal information processing, as demonstrated both *in vivo* (Haimovici et al., 2013) as well as *in vitro* (Shew et al., 2009; Tetzlaff et al., 2010).

In summary, we devise a theoretical construct that, following measurements taken at any arbitrary scale, allows for predictions to be made about how the system will behave – either as it changes in size (if scalable) or as we zoom in or out (if scale free). In this way, we create a unified approach for future studies to analyse both scalable and scale free systems via the construction of generative models within the same formal DCM-based statistical framework.

### Wide-Field Calcium Imaging

Calcium dynamics over the whole dorsal cortex of the left hemisphere are recorded using a wide-field imaging approach. Excitation light emanates from a blue LED (Thorlabs; M470L3) and is filtered (excitation filter, 480/40 nm BrightLine HC), diffused, collimated, and directed to the left hemisphere by a dichroic mirror (510 nm; AHF; Beamsplitter T510LPXRXT). The imaging system consists of two objectives (Navitar, top objective: D-5095, 50 mm f0.95; bottom objective inverted: D-2595, 25 mm f0.95). Excitation light is focused approximately 100 μm below the blood vessels. Green emission photons are collected through both objectives and dichroic, filtered (emission filter, 514/30 nm BrightLine HC) and recorded with a sensitive CMOS camera (Hamamatsu Orca Flash 4.0) mounted on top of the system. No photobleaching is observed under these imaging conditions. Images of 512 × 512 pixels are collected at 20 frames per second.

Wide-field calcium imaging through the intact skull allows for the simultaneous recording of neural population activity across the whole dorsal cortex. Although fluorescence arises from single neurons, photons are diffused through the skull. Therefore, this method does not have single-cell resolution and the measured signal represents the bulk population activity. One cubic millimetre contains approximately 10^5^ neurons and one pixel represents 43 × 43 × 150 μm (43 × 43 imaged from above). As we collect light from all labelled neurons, we take 150 μm as the total depth on layer 23, which means that one pixel represents approximately 30 neurons. However, in reality the light is diffused, which means that the DF/F values of a pixel are influenced by neighbouring pixels (Keller et al., 2018).

By spatially averaging the recorded signal, the activity of a bigger population of neurons is averaged at each coarse graining step. Additionally, hemodynamic changes may influence the signal. However, in the absence of movement (we only include periods in which the animal is sitting quietly) we have previously shown that this influence is minimal (Gilad et al., 2018). By analogy with human imaging, we might consider the fine-grained data to represent multiunit recordings from the electrocorticogram of an implanted patient, while the most coarse-grained level would be analogous to the spatially extended resting state networks. Therefore, we do not directly measure neural activity but use calcium as a proxy. This is motivated by the lack of a technique that allows the direct measurement of neural activity with similar spatiotemporal resolution as wide-field calcium imaging. Overall, this method is limited by the calcium indicator dynamics (GCaMP6f: decay τ_½_ for one action potential of ∼140 ms; rise τ_*peak*_ 1 AP ∼45 ms) and imaging speed (20 Hz) (Chen et al., 2013). However, given that the indicator is genetically encoded, we know that the signal only arises from layer 2/3 excitatory neurons.

### Sensory Mapping and Alignment

In order to align brain areas to the Allen Mouse Common Coordinate Framework (Mouse Coordinate, 2016), we perform sensory mapping under light anaesthesia (1% isoflurane) in each mouse. We present five different stimuli contralateral to the imaging side: a vibrating bar coupled to a loudspeaker is used to stimulate either (1) whiskers; (2) forelimb paw; or (3) hindlimb paw (somatosensory stimuli; 20 Hz for 2 s); (4) 2 s-long white noise is played (auditory stimulus); and (5) a blue LED positioned in front of the right eye provides a visual stimulus (100 ms duration; approximately zero elevation and azimuth). The stimuli activate a corresponding set of cortical areas. These areas, together with anatomical landmarks (Bregma; Lambda; midline; as well as the anterior, posterior, and lateral ends of the dorsal cortex), are used as anchoring points to align each individual brain to the Mouse Common Coordinate Framework. Pixels outside the borders of the Mouse Common Coordinate Framework are discarded.

### Behavioural Task

Water-deprived head-fixed mice are trained in a go/no-go auditory discrimination task with a delay. Each trial (10 s duration) commences with a trial cue (visual cue delivered by an orange LED, 1 flash, 500 ms duration) after which mice had to discriminate between two auditory tones (4 vs 8 kHz) presented for 2 s. After a delay period (2–3 s) a reward cue (3 flashes, 150 ms duration with 100 ms interval) signals the start of the response window (2 s). Pure auditory sounds are generated by a Tucker-Davis System 3 processor (RZ6) and are presented using a magnetostatic loudspeaker (MF-1, Tucker-Davis) placed ∼5 cm from the right ear (contralateral to the imaged hemisphere). Each trial is separated by an inter-trial interval of ∼5 s.

Mice are trained using the 8 kHz tone as the “go” stimulus. In order to obtain a water reward, mice have to lick a water spout in the response window during go trials (“hit”). Licks in response to the “no-go” tone are mildly punished with white noise and a time out (∼2 s, “false alarms”, FA). Licks outside the response window (“earlies”) are equally punished. The absence of licks in “no-go” (“correct-rejections”, CR) and: “go” (“misses”) trials are neither rewarded nor punished. Performance is quantified as *d*-prime: *d*′ = *Z*(Hit/(Hit + Miss))−*Z*(FA/(FA + CR)) where *Z* denotes the inverse of the cumulative distribution function. Animals are imaged upon reaching expert level performance (*d*′ > 1.5), specifically *d*′= 1.90, 2.23, and 2.36 for mouse 1, 2, and 3, respectively.

### Spontaneous Activity

We record meso-scale spontaneous activity in the same three mice that are imaged solving the task (using the same wide-field set-up) with equal trial and inter-trial interval lengths. Calcium dynamics are recorded in the absence of any external stimuli with the exception of a continuous blue light used for wide-field imaging (also present during task). This light is directed into the intact skull preparation from the optical path (placed above the heads of the mice) with an illumination intensity of <0.1 mW/mm^2^. The light in the recording environment is dim, as the light is collimated and the objective is close to the preparation.

### Movement

Although the animals are head-fixed, they are able to freely whisk and move their limbs and backs. Given the dim recording conditions, we use infrared light to monitor the animals’ movements (940 nm infra-red LED) in both states (task and spontaneous activity). We extract movement vectors of the forelimb and back region from the recordings. Movement is calculated as 1 minus frame-to-frame correlation of these two regions. We perform multiple linear regression of all recordings with respect to the animals’ movements, as well as to the external stimuli (sound and light cues) in the task recordings. It is due to this regression of movement and stimuli that we set the elements of the *C* matrix in the DCM recovery model to zero.

### Data Pre-processing

Matlab software (Mathworks) was used to pre-process the data. 512 × 512 pixel images are collected with the wide-field system and then downsampled to 256 × 256. Pixel size after downsampling was ∼40 μm. To normalize for uneven illumination or GCaMP6f expression, we calculate the percentage change of fluorescence (Δ*F*/*F*) relative to the start of each trial.

### Regions of Interest

We begin by defining two non-overlapping ROIs within the Allen Mouse Common Coordinate Framework that each span 64 × 64 pixels, as this is the largest power of 2 that can be accommodated within the imaged area. The first ROI covers (as designated by the Allen Institute) primary somatosensory areas upper and lower limb. It also includes parts of the primary and secondary motor areas; primary somatosensory area unassigned; primary somatosensory area trunk; primary somatosensory area barrel field; and the retrosplenial area. The second ROI covers the posterior parietal association areas; the anteromedial visual area; and the posteromedial visual area. It also includes parts of the primary visual area; primary somatosensory area barrel field; and primary somatosensory area trunk (see Figure 2B). Data outside the ROIs are disregarded.

### Coarse Graining

Note that we use the term “ROI” to refer to the two large areas of the cortex defined above, whereas we use the term “region” to refer to the constituents of “blocks” in the language of renormalization group theory. For each of the two ROIs, we then: (a) z-score each region’s timecourse in the 64 × 64 ROI; i.e., we subtract the mean and divide by the standard deviation on a region-wise level; (b) subdivide the 64 × 64 ROI into a grid consisting of 32 × 32 blocks; (c) run first level DCM on each of the 32 × 32 blocks, in which all connectivity matrices entered into first level DCMs are of size 2 × 2; (d) perform Bayesian model averaging on the 32 × 32 first level DCMs, such that we obtain a single representative intrinsic connectivity matrix [*A* in Eq. (14)] associated with the first scale; (e) coarse grain the 64 × 64 regions by a factor of 2 such that we obtain 32 × 32 regions, each of which corresponds to the mean of a 2 × 2 block within the original 64 × 64 ROI.

We then repeat steps (a) through (c) above for 32 × 32, 16 × 16, 8 × 8, 4 × 4, and 2 × 2 regions, each time recovering the intrinsic connectivity matrix associated with each level of coarse graining. One quarter of the blocks are randomly sampled in step (c) above for the first three scales, in the interest of computational expediency. Note that in this characterisation of coupled dynamics we are taking averages across different combinations of regions at each scale. With reference to step (d) we use a prior variance of 1, and prior means of −1 for the main diagonal and 0 for the off-diagonal coupling parameters of the *A* matrix. Therefore, we assume *a priori* that each region can be positively or negatively influenced by any other region, while maintaining stability via self-inhibition. We then enter the intrinsic connectivity matrices recovered at each level of coarse graining into the second level of the hierarchical modelling (PEB). We compare each scale to the original full-resolution 64 × 64 region data and test the extent to which the theoretical transformation in Eq. (28) holds.

## Data Availability Statement

All pre-processed data used here are made publicly available in the following repository: https://figshare.com/articles/Murine_calcium_imaging_data/12012852. DOI: Helmchen and Gallero-Salas (2020): https://doi.org/10.6084/m9.figshare.12012852.v1. The MATLAB code used to implement Eq. (7) is made available at the following public repository: github.com/allavailablepubliccode/Scaling.

## Ethics Statement

All animal experiments were carried out according to the guidelines of the Veterinary Office of Switzerland following approval by the Cantonal Veterinary Office in Zurich.

## Author Contributions

YG-S and FH collected the murine calcium imaging data. All authors designed and performed the research, analysed the data, and wrote the manuscript.

## Conflict of Interest

The authors declare that the research was conducted in the absence of any commercial or financial relationships that could be construed as a potential conflict of interest.

## Supplementary Material

Supplementary Figure 1 A sample intrinsic connectivity matrix from one trial collected in the first mouse in the spontaneous state. The six subplots show the way in which the connectivity strength (s) progresses from full resolution data (top left) to the maximum level of coarse graining used in this study (bottom right).

Supplementary Movie 1 One trial collected in the first mouse in the spontaneous state. The six subplots progress from full resolution data (top left) to the maximum level of coarse graining used in this study (bottom right) with the same colour scale used as in Figure 2B.

## Appendix 1

In the first level modelling, timecourses of the three-body system are used to recover their intrinsic connectivity via a Bayesian estimation method known as dynamic expectation maximization (DEM) (Friston et al., 2008) for each of the ten simulations. The DEM procedure seeks to estimate the “true” signal (similar to a Kalman filter). To do this, it assumes a factorizable (independent) set of unknowns in the model. The three sets are:

(1) The D-step, in which *v* and *u* are estimated with priors. In other words, the form of the ordinary differential equation (ODE) under a given *A* matrix and noise value is assumed and then the dynamic states are predicted and updated according to derivatives of a cost function (the variational free energy). The variational free energy *F* combines both accuracy and complexity when scoring models:
  $$F=\underset{\text{accuracy}}{\underbrace{\left\langle \log p\,\left(y|\theta ,m\right)\right\rangle }}-\underset{\text{complexity}}{\underbrace{K\,L\,\left[q\,\left(\theta \right),p\,\left(\theta |m\right)\right]}}$$
  where log*p*(*y*|θ,*m*) is the log likelihood of the data *y* conditioned upon model states, parameters and hyperparameters θ, and model structure *m*. This cost function combines accuracy maximisation with complexity minimisation, where complexity represents the divergence from the estimate to the prior on the trajectories of *v* and *u*.
(2) The E-step, in which the model parameters are optimised as represented in the *A* matrix – these are updated according to derivatives of the free energy.
(3) The M-step, in which the magnitude of random fluctuations (known as hyperparameters) are updated (again based on their free energy derivatives) while holding the other sets constant. Overall this scheme should converge (after repetition of D, E, and M steps) on a posterior probability of all three sets of unknowns.

## Appendix 2

In the second level modelling, we use a hierarchical Bayesian scheme to assess the degree to which changes in intrinsic connectivity matrix elements can be explained by the theoretically predicted transformation for: (a) scalable systems for a range of α values recovered for different sized orbits; and (b) scale free systems for a range of dynamical critical exponents *z* recovered for different levels of coarse graining in neuroimaging data.

This statistical test uses a parametric empirical Bayesian (PEB) approach – which allows us to test over multiple individual models (e.g., at different scales) and to define between-model covariates of interest. In other words, PEB provides a way to perform a test similar to a general linear model (GLM) or regression and to account for posterior densities, rather than just point estimates (since the DCMs return full probability distributions). Here we construct a set of potential between-model tests that account for different possible changes in both scalable and scale free systems.

Parametric empirical Bayesian was repeated for each plausible value of (a) α in the context of the orbital mechanics analysis, and (b) the dynamical critical exponent *z* in the context of the neuroimaging analysis – (both in steps of 0.005). These line searches enabled us to track the free energy approximation to model evidence (as well as the posterior expectation of second-level parameters) as a function of (a) α for the orbital mechanics analysis and (b) *z* for the neuroimaging analysis. We use the model *Y* = *X*β + ∈, where *Y* is a column vector comprising DCM estimates (means and variances) at different scales and *X* is a column vector which contains the theoretically determined scale – specified by the scaling parameter *b*. PEB returns the approximate model evidence (free energy), as well as the posterior over β for each value of between-model tests. This allows us to determine the best possible value of (a) α (for the physical scaling of the orbits) or (b) *z* (for the coarse graining of the images) that would account for the changes in the individual DCMs. In the case of the orbital analysis, we repeat the hierarchical PEB scheme 100 times, each time adding Gaussian noise to the scaling parameter *b*, such that we obtain a range of maximum free energies. We are therefore in a position to test the hypothesis that the variation in connectivity with respect to scale in Eq. (14) is best explained using a scaling exponent value close to α = −32 in accordance with Kepler’s third law in Eq. (6).

## Appendix 3

### Animals and Surgical Procedures

We use three triple transgenic Rasgrf2-2A-dCre; CamK2a-tTA; and TITL-GCaMP6f adult male mice (3–5 months old). This line is characterised by inducible, specific and high expression of the calcium indicator GCaMP6f in pyramidal layer 2/3 neurons of the neocortex (Madisen et al., 2015). To induce the expression of the indicator, destabilized Cre must be stabilized by trimethoprim (TMP). Individual mice are intraperitoneally injected with 150 μg TMP/g of body weight reconstituted in Dimethyl sulfoxide (DMSO, Sigma 34869) at a saturation level of 100 mg/ml.

In order to expose the skull above the left brain hemisphere for wide-field calcium imaging, we use a minimally invasive intact skull preparation technique. Briefly, mice are anaesthetized (2% isoflurane in pure O_2_) and their temperature controlled (37°C). After removing the skin and connective tissue above the dorsal skull, we clean and dry the skull. We then apply a layer of UV-cure iBond over the skull, followed by a second layer of transparent dental cement (Tetric EvoFlow T1). Dental cement “worms” (Charisma) are applied around the preparation and a metal head post for head fixation is glued to the preparation. The resulting imaging window ranges from ∼3 mm anterior to bregma to ∼1 mm posterior to lambda and ∼5 mm laterally to midline.
