# Supplementary figures and images for: Neural Systems Under Change of Scale
Source: Front Comput Neurosci. 2021 Apr 21;15:643148. doi: 10.3389/fncom.2021.643148 (PMC8099030; doi:10.3389/fncom.2021.643148)

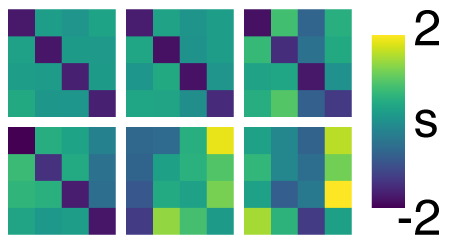

Supplement: Supplementary Figure 1 — A sample intrinsic connectivity matrix from one trial collected in the first mouse in the spontaneous state. The six subplots show the way in which the connectivity strength (s) progresses from full resolution data (top left) to the maximum level of coarse graining used in this study (bottom right). [file Image_1.tif]
